# A comparative biochemical investigation of the impeding effect of C1-oxidizing LPMOs on cellobiohydrolases

**DOI:** 10.1016/j.jbc.2021.100504

**Published:** 2021-03-03

**Authors:** Malene Billeskov Keller, Silke Flindt Badino, Nanna Røjel, Trine Holst Sørensen, Jeppe Kari, Brett McBrayer, Kim Borch, Benedikt M. Blossom, Peter Westh

**Affiliations:** 1 Department of Geosciences and Natural Resource Management, University of Copenhagen, Frederiksberg, Denmark; 2 Department of Biotechnology and Biomedicine, Technical University of Denmark, Lyngby, Denmark; 3 Novozymes A/S, Lyngby, Denmark; 4 Novozymes, Inc, Davis, California, USA

**Keywords:** cellobiohydrolase, cellulose, cellulase, enzyme kinetics, pre-steady-state kinetics, glycoside hydrolase family 7, synergism, lytic polysaccharide monooxygenase (LPMO), cellulase cocktails, AA, auxiliary activity, BCA, bicinchoninic acid, CDH, cellobiose dehydrogenase, DP, degree of polymerization, DS, degree of synergy, GH, glycoside hydrolase, HPAEC-PAD, high-performance anion-exchange chromatography with pulsed amperometric detection, LPMO, lytic polysaccharide monooxygenase, PAHBAH, p-hydroxybenzoic acid hydrazide, PASC, phosphoric acid swollen cellulose

## Abstract

Lytic polysaccharide monooxygenases (LPMOs) are known to act synergistically with glycoside hydrolases in industrial cellulolytic cocktails. However, a few studies have reported severe impeding effects of C1-oxidizing LPMOs on the activity of reducing-end cellobiohydrolases. The mechanism for this effect remains unknown, but it may have important implications as reducing-end cellobiohydrolases make up a significant part of such cocktails. To elucidate whether the impeding effect is general for different reducing-end cellobiohydrolases and study the underlying mechanism, we conducted a comparative biochemical investigation of the cooperation between a C1-oxidizing LPMO from *Thielavia terrestris* and three reducing-end cellobiohydrolases; *Trichoderma reesei* (*Tr*Cel7A), *T. terrestris* (*Tt*Cel7A), and *Myceliophthora heterothallica* (*Mh*Cel7A). The enzymes were heterologously expressed in the same organism and thoroughly characterized biochemically. The data showed distinct differences in synergistic effects between the LPMO and the cellobiohydrolases; *Tr*Cel7A was severely impeded, *Tt*Cel7A was moderately impeded, while *Mh*Cel7A was slightly boosted by the LPMO. We investigated effects of C1-oxidations on cellulose chains on the activity of the cellobiohydrolases and found reduced activity against oxidized cellulose in steady-state and pre-steady-state experiments. The oxidations led to reduced maximal velocity of the cellobiohydrolases and reduced rates of substrate complexation. The extent of these effects differed for the cellobiohydrolases and scaled with the extent of the impeding effect observed in the synergy experiments. Based on these results, we suggest that C1-oxidized chain ends are poor attack sites for reducing-end cellobiohydrolases. The severity of the impeding effects varied considerably among the cellobiohydrolases, which may be relevant to consider for optimization of industrial cocktails.

Saprotrophic fungi secrete an array of enzymes enabling the deconstruction of cellulosic biomass. These enzymes usually work in synergy, meaning that they show higher activity when acting together compared with the sum of their activities when working separately. Synergy among cellulolytic enzymes is crucial for the degradation of lignocellulosic material in nature and in industrial settings such as in biorefineries that convert biomass into fuel and materials.

In the classical scheme for enzymatic cellulose degradation, cellulose is hydrolyzed by three main classes of glycoside hydrolases; exo-acting enzymes (cellobiohydrolases), endo-acting enzymes (endoglucanases), and β-glucosidases that catalyze the hydrolysis of soluble oligosaccharides to glucose. Recently, a new class of cellulose-active enzymes, lytic polysaccharide monooxygenases (LPMO) from Auxiliary Activity family 9 (AA9), were reported (1, 2, 3, 4, 5, 6). In contrast to hydrolases, AA9s employ an oxidative mechanism to cleave cellulose chains while oxidizing one of the carbons in the scissile bond.

Preceding the elucidation of the catalytic mechanism, patents indicated that AA9s (at that time categorized as GH61) could boost the activity of glycoside hydrolases (7, 8), and today the ability of LPMOs to boost the efficiency of cellulase cocktails is widely recognized and LPMOs are crucial components of cellulolytic cocktails (2, 4, 6, 9, 10, 11, 12, 13). Several mechanisms of the synergistic cooperation between glycoside hydrolases and LPMOs have been proposed. LPMOs have been shown to bind to crystalline regions in the substrate and cause fibrillation of the cellulose chains, which is suggested to enhance the number of adsorption sites for glycoside hydrolases (14, 15, 16, 17, 18). Vermaas and colleagues studied the synergistic effect between LPMOs and cellobiohydrolases using molecular dynamics simulations and suggested that the main role of LPMOs is to create solvent-accessible ends for glycoside hydrolases (19). LPMOs have also been reported to enhance the processive mobility of cellobiohydrolases (15). Hu and colleagues reported that additions of LPMO increased the desorption of a reducing-end cellobiohydrolase and suggested that addition of AA9 may enhance the hydrolysis by releasing unproductively bound cellobiohydrolases (20).

In contrast to the common perception of synergistic effects between LPMOs and glycoside hydrolases, a few studies have reported pronounced impeding effects of C1-oxidizing LPMOs on the activity of GH7 (reducing end) cellobiohydrolases from *Trichoderma* spp. (17, 21, 22, 23). This may have important implications, as GH7 cellobiohydrolases make up a significant part of industrial cellulolytic cocktails (24). To elucidate whether the impeding effect of C1-oxidizing LPMOs is generic for GH7 cellobiohydrolases and to shed light on the mechanism behind the impeding effect, we have conducted a comparative biochemical study of the cooperation between a C1-oxidizing LPMO and three GH7 cellobiohydrolases. Bioinformatics has revealed that the distribution of AA9 varies considerably across species, from a few genes in *Trichoderma* spp. to 40 genes in other plant cell-wall-degrading fungi (25, 26). This may suggest that fungi employ different enzymatic strategies, which could be relevant to consider when investigating the cooperation between enzymes from different fungi. In light of this, we selected the cellobiohydrolases to represent hosts with varying numbers of putative AA9s in their genome; *Trichoderma reesei*, with 2 (26), *Thielavia terrestris* with 18, and *Myceliophthora heterothallica* with 22 (25, 27). The three cellobiohydrolases were heterogeneously expressed in the same expression organism and thoroughly characterized biochemically. The three cellobiohydrolases contain a C-terminal carbohydrate-binding module type 1 (CBM1) and share 56% sequence identity (*Tr*Cel7A and *Mh*Cel7A share 60% identity, *Tr*Cel7A and *Tt*Cel7A share 64% identity, and *Mh*Cel7A and *Tt*Cel7A share 78% identity). We investigated the synergistic effect between the LPMO and the cellobiohydrolases in terms of the degree of synergy (DS). In addition, we investigated how oxidations on cellulose chains influence the Michaelis–Menten kinetics, pre-steady-state kinetics, and rate of substrate complexation of the three cellobiohydrolases. These results contribute to our understanding of enzyme cooperation in cellulolytic cocktails and may be relevant for optimization of industrial cocktails with higher degrees of synergy.

## Results

### Synergistic effects between the cellobiohydrolases and the AA9

**Figure 1:**
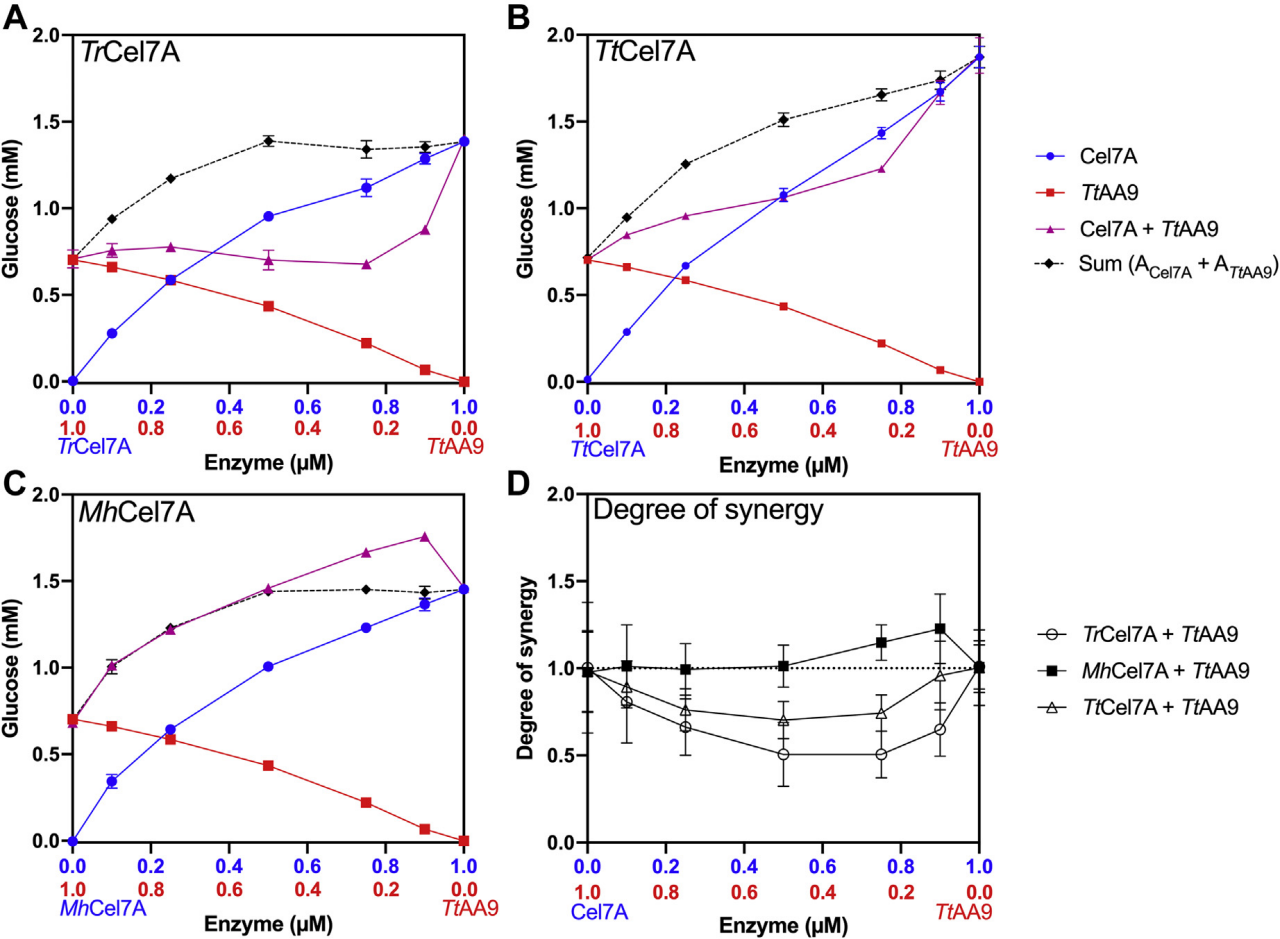
**Synergy curves.** Glucose formation by the three cellobiohydrolases (*A*, *Tr*Cel7A, *B*, *Tt*Cel7A, *C Mh*Cel7A) (*blue circles*), the LPMO (*red squares*), and the combined cellobiohydrolase and LPMO (*purple triangles*) against Avicel (10 g/L). In the experiments with the monocomponents, the enzyme concentration was varied (0–1 μM). In the experiments with cellobiohydrolase and LPMO, the enzyme concentration was constant (1 μM) with varying enzyme ratios. The *black diamonds* and the *dotted line* indicate the sum of the glucose formation by the monocomponents. Symbols are averages of triplicate measurements, and *error bars* represent standard deviations (SD). *D*, degree of synergy (DS) calculated according to Equation 1 for pairs of cellobiohydrolase and *Tt*AA9 (*Tr*Cel7A *open circles*, *Tt*Cel7A *open triangles*, *Mh*Cel7A *squares*), plotted as a function of varying enzyme ratios. The *dotted line* represents DS = 1. *Error bars* are SD propagated forward from original SD from *A*–*C*. *Lines* between symbols are only shown to guide the eye.

The activity of the LPMO, *Tt*AA9, and the three cellobiohydrolases, *Tr*Cel7A, *Tt*Cel7A, and *Mh*Cel7A, was assessed in 3-h experiments with varying enzyme concentrations from 0 to 1 μM. The activity of the cellobiohydrolases in combination with *Tt*AA9 was also assessed from 3-h experiments with varying enzyme ratios and a constant total enzyme concentration of 1 μM. After the reaction, β-glucosidase was added to the supernatant to hydrolyze the soluble products to glucose. Figure 1, *A–C* illustrate the activity of the cellobiohydrolase (blue), the AA9 (red), and the combined cellobiohydrolase and AA9 (purple). The concentration of cellobiohydrolase is increasing from left to right, whereas the concentration of AA9 is increasing from right to left. The sum of the individual activities of the cellobiohydrolase and the AA9 is represented with a dotted line and black diamonds. Figure 1*D* illustrates the degree of synergy, that is, the activity of the mixture of the two enzymes (A_Cel7A+AA9_) divided by the sum of their individual activities (A_Cel7A_ +A_AA9_),

(1) $$\text{DS}=\frac{{\text{A}}_{\left(\text{Cel}7\text{A}+\text{AA}9\right)}}{{\text{A}}_{\text{Cel}7\text{A}}+{\text{A}}_{\text{AA}9}}$$

A DS >1 indicates that the components act synergistically, while DS < 1 indicates impediment. Negative synergy (DS < 1) or impediment was observed for the combination of *Tr*Cel7A+AA9 with DS between 0.5 and 0.8. The data in Figure 1*A* show that the activity of the mixture (*Tr*Cel7A + AA9) at the lowest ratio of AA9 to Cel7A (10%:90%) resulted in 35% less glucose than that of 100% *Tr*Cel7A, indicating a pronounced impeding effect.

The combination *Tt*Cel7A+ AA9 also showed impediment, although to a lesser extent than for *Tr*Cel7A + AA9, with DS varying from 0.7 to 1. At the lowest ratio of AA9 to Cel7A (10%:90%), the DS was approximately 1, suggesting additivity. A different pattern was observed for the combination *Mh*Cel7A + AA9, for which the DS varied between 1 and 1.2, indicating no synergy or slight synergy.

### Activity of cellobiohydrolases against oxidized cellulose

To investigate the origin of the impeding effect between the cellobiohydrolases and the LPMO, we chemically oxidized the cellulose substrate at the C1-position on the chain ends (the reducing ends) to mimic LPMO-induced oxidations. The insoluble reducing ends in phosphoric acid swollen cellulose (PASC) were oxidized in a redox reaction in which Cu(II) is reduced to Cu(I) and the reducing ends, *i.e.*, the aldehydes, are oxidized to aldonic acids. A control experiment in which cellotetraose was incubated with the Cu(II)-BCA reaction mixture verified that the oxidation method did not introduce chain breakage (Fig. S1), which is in line with the results of previous studies (28, 29). As described in Materials and Methods, the level of oxidation was determined by two approaches. The level of oxidation of the reducing ends was determined to be 39.8 ± 0.3% by the first approach and 33.0 ± 0.5% by the second.

**Figure 2:**
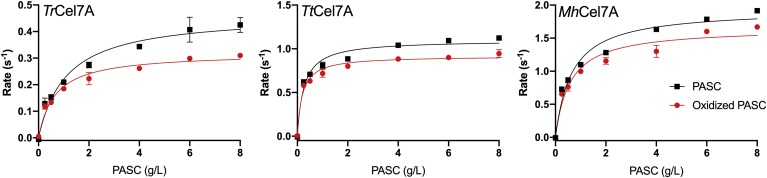
**Michaelis–Menten curves.** Specific rate of *Tr*Cel7A, *Tt*Cel7A, and *Mh*Cel7A against varying loads of PASC (*black squares*) or oxidized PASC (*red circles*) for 1 h experiments. Symbols are averages of triplicate measurements, and *error bars* represent standard deviations (SD). *Lines* are best fit of the Michaelis–Menten equation (Equation 2).

Figure 2 shows the specific activity of the three cellobiohydrolases against varying loads of PASC and oxidized PASC analyzed with respect to the Michaelis–Menten equation,

(2) $$v=\frac{V_{\max}s_{o}}{K_{m}+s_{o}}$$

where *v* is the steady-state rate, and *S*_*0*_ is the initial substrate load in g/L. The results show that the maximal rate at substrate saturation, V_max_, and the Michaelis–Menten constant, K_M_, were lower on oxidized cellulose compared with nonoxidized cellulose. For *Tr*Cel7A, V_max_ and K_M_ were approximately 30% and 40% lower, respectively, on oxidized PASC compared with the nonoxidized PASC. The effect was less pronounced for *Tt*Cel7A and *Mh*Cel7A, for which V_max_ was approximately 15% lower on the oxidized substrate, and K_M_ was approximately 15% and 25% lower on the oxidized substrate, respectively. The parameters are listed in Table 1.

**Table 1:** Parameters from Michaelis–Menten experiments, processivity experiments, and real-time measurement of complexation

Cel7A	Substrate	V_max_/E_0_ (s^−1^)	K_M_ (g L^−1^)	n	*k*_*on*_ (L/g s^−1^)	K_d_ (gL^−1^) × 10^−3^	*k*_*off*_ (s^−1^) × 10^−3^
*Tr*Cel7A	PASC	0.47 ± 0.02	1.11 ± 0.15	9.5 ± 0.4	1.97 ± 0.07	33.33 ± 5.52	65.76 ± 11.17
	Oxidized PASC	0.32 ± 0.01	0.65 ± 0.07	8.4 ± 0.2	1.53 ± 0.04	37.99 ± 3.60	58.12 ± 5.72
*Tt*Cel7A	PASC	1.10 ± 0.02	0.26 ± 0.03	15.7 ± 0.4	1.55 ± 0.03	20.29 ± 2.13	31.45 ± 3.36
	Oxidized PASC	0.92 ± 0.02	0.19 ± 0.02	15.8 ± 0.7	1.31 ± 0.02	24.56 ± 3.75	32.12 ± 4.94
*Mh*Cel7A	PASC	1.94 ± 0.06	0.65 ± 0.08	10.7 ± 0.3	1.85 ± 0.06	24.43 ± 2.59	45.17 ± 5.04
	Oxidized PASC	1.65 ± 0.05	0.58 ± 0.08	10.1 ± 0.5	1.67 ± 0.01	35.08 ± 2.58	58.55 ± 4.33

We also studied the effect of substrate oxidation in experiments with low substrate load and varying enzyme concentration until saturation, that is, so-called inverse Michaelis–Menten experiments (30) (Fig. S2). Under these conditions, the activity against the two substrates was almost identical for all three cellobiohydrolases.

**Figure 3:**
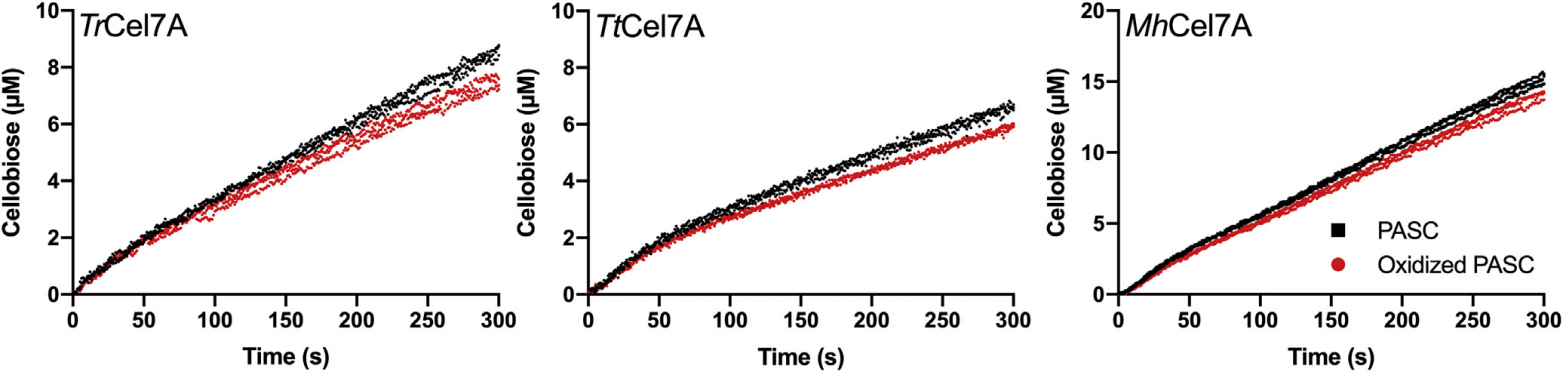
**Real-time recording of hydrolytic activity against P ASC) and oxidized PASC.** Cellobiose production of 100 nM *Tr*Cel7A (*left graph*), *Tt*Cel7A (*middle graph*), and *Mh*Cel7A (*right graph*) against 1 g/L PASC (*black squares*) or oxidized PASC (*red circles*) plotted against time. The experiments were performed in triplicates, and each symbol represents a replicate measurement.

The effect of substrate oxidation was further studied in real-time measurements of the progress curves using a CDH biosensor (31). The data in Figure 3 show the cellobiose formation of each of the cellobiohydrolases within the first 5 min of the reaction. The results showed that the activity of all three cellobiohydrolases was lower on the oxidized cellulose than on the nonoxidized cellulose. The rates of cellobiose accumulation between 100 and 300s, derived as the slope of linear regressions, were 14 ± 0.7, 10 ± 0.5, and, 7 ± 0.4 % lower on the oxidized cellulose than on the nonoxidized cellulose for *Tr*Cel7A, *Tt*Cel7A, and *Mh*Cel7A, respectively.

The effect of the substrate oxidation on the activity of the cellobiohydrolases was further studied by analyzing the product profiles of the cellulases on the two substrates. As cellobiose is the main product of processive hydrolysis, whereas glucose and cellotriose are primary products released at initial hydrolysis events, processivity can be assessed as the ratio of cellobiose/(glucose + cellotriose) (32, 33, 34). Using this approach, we found similar processivity numbers for *Tt*Cel7A and *Mh*Cel7A on the two substrates, whereas the processivity number of *Tr*Cel7A was approximately 12% lower on oxidized cellulose compared with the nonoxidized cellulose (Fig. S3). The processivity numbers, n, are listed in Table 1.

### Rate of complexation and decomplexation of the cellobiohydrolases on PASC and oxidized PASC

The effect of substrate oxidation on the activity of the three cellobiohydrolases was further investigated by a fluorescence-based method that detects ligand–enzyme complexation (35). The substrate-binding tunnel of cellobiohydrolases is decorated with tryptophan residues that interact with the cellulose chain (36). The intrinsic fluorescence signal of these tryptophan residues will increase upon cellulose binding (37, 38). The increase in signal is likely a result of dequenching as water molecules are removed from the indole side chains as the cellulose chain is bound in the substrate-binding tunnel.

**Figure 4:**
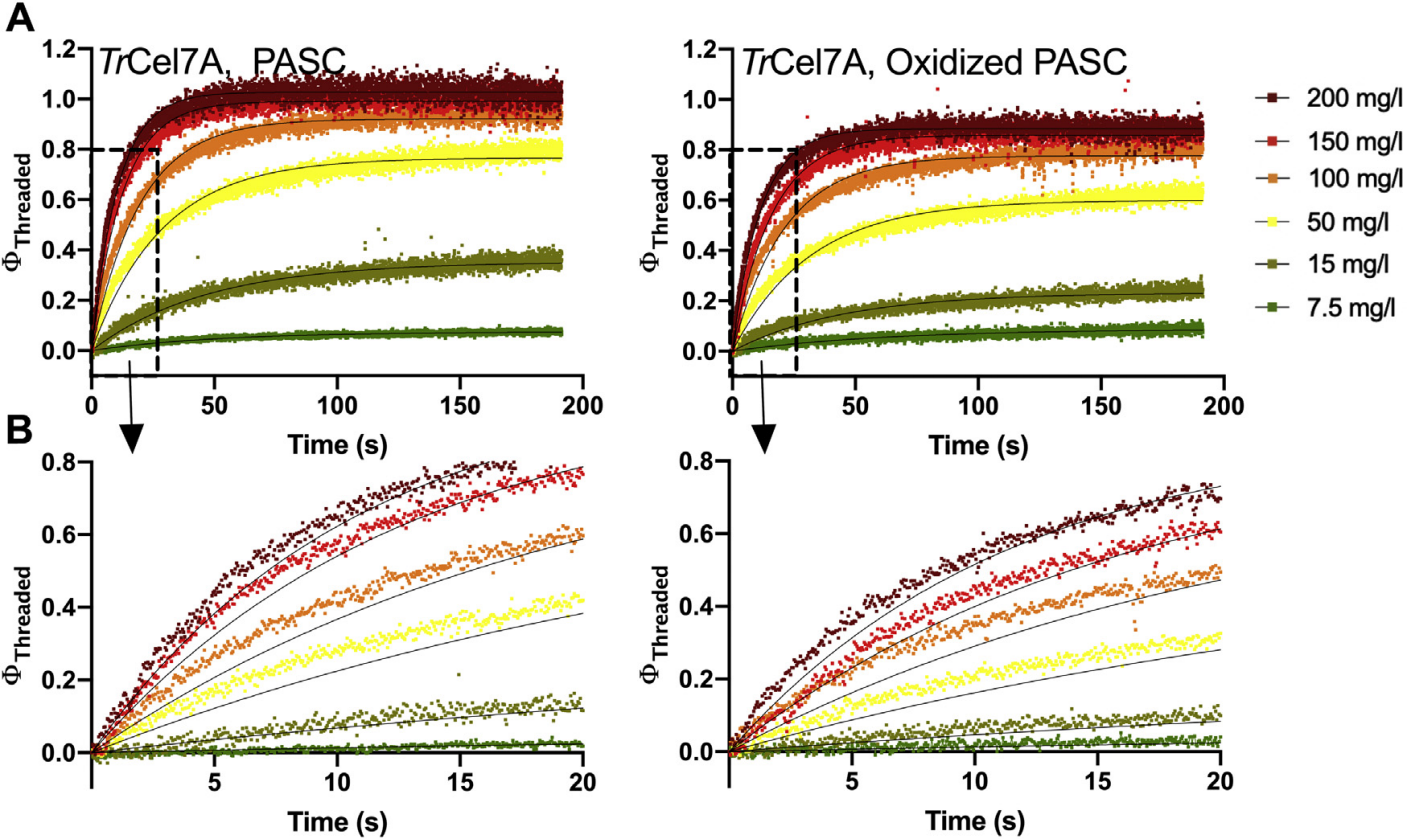
**Real-time fluorescence data for the complexation of phosphoric acid swollen cellulose (PASC) and oxidized PASC.***A*, real-time fluorescence data for the complexation of various loads of PASC (*left*) and oxidized PASC (*right*) of *Tr*Cel7A represented as the fraction of threaded enzymes, Φ_threaded_ against time. The lines represent the best fit to an exponential function of the type, F_x_(t)=F_eq_(1 − e^−*k*t^). B. Enlargements of the data from 0 to 20 s, as indicated by the *dotted boxes* in *A*.

The change in fluorescence was recorded in samples with each of the three cellobiohydrolases after the injection of substrate. These experiments were repeated with varying loads of cellulose and oxidized cellulose. Figure 4 shows the fraction of *Tr*Cel7A (similar data for *Mh*Cel7A and *Tt*Cel7A can be found in Fig. S4) in an enzyme–substrate complex as a function of time after injection of substrate. The fraction of enzymes in a substrate complex, Φ threaded, was calculated based on the increment in the fluorescence signal at saturation (where *E*_0_ ∼ [ES]), *F*_max_ as,

(3) $$\Phi_{threaded}=\frac{\left[ES\right]}{E_{o}}=\frac{F_{x}}{F_{\max}}$$

where *F*_x_ is the increment in signal in an experiment with a given cellulose load (35).

The curves were fitted to exponential functions of the type F_x_(t)=F_eq_(1 − e^−*k*t^), where F_x_(t) is the real-time fluorescence signal, and F_eq_ is the equilibrium fluorescence signal, *i.e.*, the plateau level for each substrate load.

The method has a dead time of a few hundred milliseconds (35), and this interval was thus ignored in the data analysis. The method allows for simple determination of the on-rate, *v*_*on*_, as the initial slope of the fluorescence signal. The initial slopes were determined as the mathematical derivative of the fitted exponential functions for t→0.

The interpretation of the data is based on the assumption that the substrate oxidation does not affect the fluorescence signal of the enzyme–substrate complex. A previous study has shown that the tryptophan at residue 38 (W38) makes a substantial contribution to the increase in fluorescence signal for *Tr*Cel7A (35). This residue is situated in the middle of the catalytic tunnel of Cel7A, approximately 20–25 Å from the reducing end. Thus, we assume that the increase in fluorescent signal upon substrate binding is independent of the oxidation and instead represents the number of substrate-bound tryptophan residues, in particular W38.

**Figure 5:**
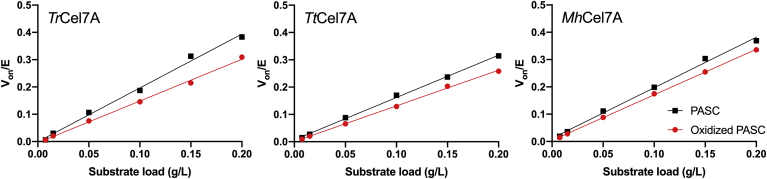
**Initial rate of complexation as a function of substrate load.** Initial rate of complexation V_on_/E_0_, of *Tr*Cel7A, *Tt*Cel7A, and *Mh*Cel7A, derived from the time course experiments as shown for *Tr*Cel7A in Figure 4 (and in Fig. S4 for *Mh*Cel7A and *Tt*Cel7A), plotted as a function of the load of PASC (*black squares*) or oxidized PASC (*red circles*). *Error bars* represent standard deviations (SD). The *lines* represent best fit of linear regressions, and the slope of each regression signifies the on-rate constant, *k*_*on*_.

The data in Figure 5 show initial rates of complexation, *v*_*on*_/E_0_, for *Tr*Cel7A, *Tt*Cel7A, and *Mh*Cel7A on PASC and oxidized PASC as a function of substrate load. The results show that *v*_*on*_/E_0_ scaled linearly with the substrate load. It follows that the on-rate constants, *k*_*on*_, can be assessed as the slope of the best-fit line to the experimental data. It directly appears from Figure 5 that the on-rate constants were lower on the oxidized cellulose compared with the nonoxidized cellulose for all three cellobiohydrolases. The on-rate constant *k*_*on*_ of *Tr*Cel7A, *Tt*Cel7A, and *Mh*Cel7A was approximately 20%, 15%, and 10% lower on the oxidized substrate compared with the nonoxidized substrate, respectively.

The dissociation constant, K_d_, was determined as the half-saturation constant of a plot with the fluorescence equilibrium (F_eq_) against the substrate load as described elsewhere (Fig. S5) (35). Finally, the off-rate constant *k*_*off*_ could be derived from the relationship K_d_ = *k*_*off*_/*k*_*on*_. The parameters of the three cellobiohydrolases on oxidized and nonoxidized PASC are listed in Table 1.

The standard error on the derived parameter, K_d_, and hence *k*_*off*_ was relatively high compared with the difference between the obtained parameters. For this reason, we cannot draw any clear conclusions based on these parameters. However, no systematic trend *k*_*off*_ was detected for the two substrates.

**Figure 6:**
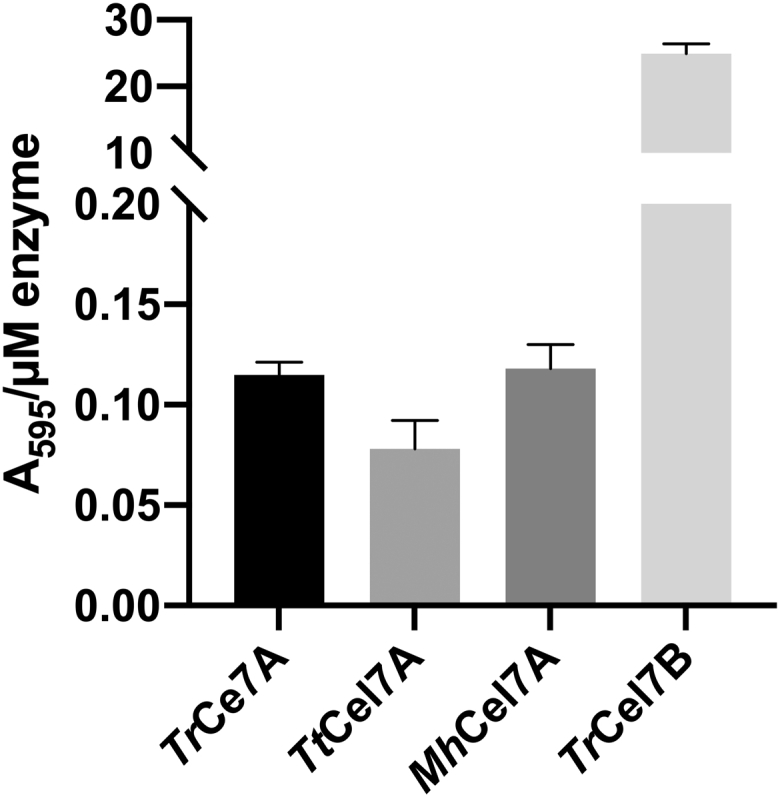
**Activity per μM enzyme (arbitrary units) of the three cellobiohydrolases and an endoglucanase for comparison (*Tr*Cel7B) on azurine-cross-linked cellulose (AZCL-HE-Cellulose)**. The activity was estimated from absorption at 595 nm (A_595_) after 2 h hydrolysis and reported as A_595_/E_0_, where E_0_ is the enzyme concentration in the assay. The substrate load was 3 g/L, and the enzyme concentrations were 5 μM for the cellobiohydrolases and 0.1 μM for the endoglucanase. Each bar represents the average of triplicate measurements, and *error bars* represent standard deviations (SD).

The results in Figure 2, Figure 3, Figure 4, Figure 5 showed that C1-oxidations affected the activity and rate of complexation of the cellobiohydrolases to different degrees. To investigate if these differences could be related to the frequency of endolytic attacks, we studied the activity of the three cellobiohydrolases on azurine-cross-linked cellulose. The results in Figure 6 show the activity of the three cellobiohydrolases and an endoglucanase *Tr*Cel7B for comparison against azurine-cross-linked cellulose. As expected, the endoglucanase showed much higher activity (240-fold) on this substrate than the cellobiohydrolases. The results revealed only moderate differences between the three cellobiohydrolases, with the lowest activity observed for *Tt*Cel7A while *Tr*Cel7A and *Mh*Cel7A showed similar activities against azurine-cross-linked cellulose.

## Discussion

While the boosting effect of LPMOs is well documented on cellulase cocktails, pronounced impeding effects have been reported between C1-oxidizing LPMOs and reducing end-cellobiohydrolases from *Trichoderma* spp. (17, 21, 22, 23). This may be of high industrial relevance as GH7 cellobiohydrolases are often the most prevalent members of fungal cellulolytic cocktails (24). The mechanism for the impeding effect has never been investigated, but different interpretations have been put forward. Current suggestions that could explain the impeding effect are: enzyme–enzyme competition for sites (17), enzymes blocking the activity of each other, oxidative inactivation (21, 39), and inability or reduced ability of the cellobiohydrolases to attack C1-oxidized cellulose chain ends (22, 23). In this present study, we investigated the mechanism behind this undesirable effect. Furthermore, we investigated whether the impeding effect of C1-oxidizing AA9s is limited to a few cellobiohydrolases or is generic for GH7 cellobiohydrolases. The three cellobiohydrolases from *T. reesei*, *M. heterothallica,* and *T. terrestris* were heterogeneously expressed in the same host organism and thoroughly characterized biochemically. The synergistic effect between the LPMO and the cellobiohydrolases was systematically investigated in terms of their degree of synergy (DS). Furthermore, we investigated the effect of oxidations on the substrate on the Michaelis–Menten kinetics, pre-steady-state kinetics, and rate of substrate complexation of the three cellobiohydrolases.

### LPMO–cellobiohydrolases synergy

The data in Figure 1 show that the synergistic effect between the LPMO and each of the three GH7 cellobiohydrolases varied noticeably. Neutral or slight synergistic effects were observed for the pair *Mh*Cel7A+ *Tt*AA9 with DS values of 1–1.2. In contrast, impeding effects were observed for the pairs *Tr*Cel7A+ *Tt*AA9 and *Tt*Cel7A+ *Tt*AA9. The pair *Tr*Cel7A+ *Tt*AA9 showed pronounced impeding effects at all Cel7A: *Tt*AA9 ratios tested with DS values in the range 0.5–0.8. This is similar to DS values previously published for the same enzyme pair (22). The impeding effect was less pronounced for the pair *Tt*Cel7A+ *Tt*AA9 with DS values of 0.7–1.

Previous reports of impeding effects between C1-oxidizing LPMOs and cellobiohydrolases are based on experimental data with GH7 cellobiohydrolases from *Trichoderma* spp. (17, 21, 22, 23). The data in Figure 1 show that the impeding effect between GH7 cellobiohydrolases and C1-oxidizing LPMOs is not limited to cellobiohydrolases from the AA9 poor *Trichoderma* spp. and that impeding effects may also occur when both the C1-oxidizing LPMO and the GH7 cellobiohydrolase are from the same organism as for the pair *Tt*Cel7A and *Tt*AA9.

### Cellobiohydrolase activity against oxidized cellulose

To investigate the mechanism behind the impeding effect, we studied how chemical cellulose oxidation at the C1-position affected the kinetics of cellobiohydrolases. We performed Michaelis–Menten experiments (Fig. 2), *i.e.*, experiments with low enzyme concentration and varying substrate loads until saturation and inverse Michaelis–Menten experiments (30) (Fig. S2), *i.e.*, experiments with low substrate load and varying enzyme concentrations until saturation. Furthermore, we studied the cellobiose formation in real-time pre-steady-state measurements (Fig. 3). The data in Figures 2 and 3 consistently showed that the activity of the cellobiohydrolases, assessed as the maximal velocity or the cellobiose production, was lower on the oxidized substrate than on the nonoxidized substrate. In contrast to the results of the Michaelis–Menten experiments and the pre-steady-state measurements, we observed almost identical inverse Michaelis–Menten curves on the oxidized substrate compared with that on the nonoxidized substrate (Fig. S2). When comparing the decrease in V_max_/E_0_ and the decrease in cellobiose production after 5 min contact time (Fig. 3), we see that the impeding effect of the cellulose oxidation scaled with the synergy results from Figure 1. *Tr*Cel7A, the cellobiohydrolase that showed the most pronounced impeding effect with the LPMO (DS ∼ 0.5–0.8 in Fig. 1), was also the cellobiohydrolase that was most negatively affected by the chemical oxidation. Furthermore, *Mh*Cel7A that showed no or slight synergy with the LPMO was least affected by the oxidations. The scaling between these independent experiments is interesting since they were made in the presence or absence of LPMOs, applying two different cellulose substrates (Avicel or PASC) and timescales. This is an indication that the impeding effect of C1-oxidizing LPMOs on the activity of GH7 cellobiohydrolases is related to the LPMO-induced oxidations on the substrate and not to the presence of the LPMO itself. Hence, we propose that C1-oxidizing LPMOs create poor attack sites for the cellobiohydrolase. Kinetic investigation of three cellobiohydrolases shows that the magnitude of the impeding effect differs but arises from a lower complexation rate. We cannot rule out other effects such as enzyme–enzyme competition for sites, enzymes blocking the activity of each other, or inactivation by LPMO generated H_2_O_2_, but our result shows that decreased complexation rate on C1-oxidized ends is an important part of the observed impeding effect.

In addition to C1-oxidation as studied here, oxidation at the C6- and C4-position of the glucosyl units may also affect the activity of cellulases (40, 41, 42). A recent study reported that *Tr*Cel7A shows synergy with the C1−C4 oxidizing LPMOs, *Thermoascus aurantiacus* AA9A, and *Lentinus similis* AA9A, while impeding effects were observed for *Tr*Cel7A and the C1-oxidizing *Tt*AA9 (22). This indicates that the impeding effect of LPMOs on reducing-end cellobiohydrolases is specific for C1-oxidizing LPMOs. However, chemical oxidation of the hydroxyls at the C6-position of the glucosyl units has been reported to impede the activity of cellulases. A study by Kato *et al*. (40) showed that the reducing-end cellobiohydrolase from *Trichoderma viride* is unable to hydrolyze C6-oxidized cellulose. Other studies have shown that oxidations at the C6-position severely impede the activity of cellulase mixtures (41, 42).

### Functional breadth among the three cellobiohydrolases

In addition to the Michaelis–Menten analysis and the pre-steady-state kinetics, we also studied the rate of substrate complexation and decomplexation (Figs. 4 and 5; Figs. S5 and S6). The kinetic parameters of all analyses are presented in Table 1. The data allow direct comparison of kinetic parameters of the three wild-type cellobiohydrolases as well as direct comparison of the effect of substrate oxidation on the kinetic parameters.

Before assessing the effect of substrate oxidation, we will compare the functional data of the three wild-type cellobiohydrolases against PASC. The data revealed substantial variability (4-fold) in both V_max_ and K_M_ for the three cellobiohydrolases. A previous study elucidating functional breadth among GH6 cellobiohydrolases revealed similar variances in these two parameters (43). In contrast to the marked differences observed in V_max_ and K_M_, the rates of substrate complexation and decomplexation showed less variability. The on-rate, *k*_*on*_ varied between 1.3 and 2 (g/L)^−1^ s^−1^, which is similar to previously published rates of substrate complexation for *Tr*Cel7A against amorphous cellulose (44). The off-rate, *k*_*off*_ varied in the range 0.03–0.07 s^−1^, which is in good agreement with recent measurements of the off-rate of *Tr*Cel7A based on single-molecule fluorescence imaging (45).

### Effect of oxidized cellulose on the kinetics of cellobiohydrolases

We will now turn the focus to the effect of substrate oxidation of the kinetic parameters of the three cellobiohydrolases. The data in Table 1 showed that substrate oxidation led to reduced V_max_, K_M_, and *k*_*on*_ off all cellobiohydrolases. Although a clear conclusion regarding *k*_*off*_ was challenged by a high standard deviation on the derived parameter, we did not observe any clear change in *k*_*off*_ by the oxidation of the substrate. The rates of substrate complexation and decomplexation listed in Table 1 support previous studies that have suggested that the rate of hydrolysis by cellobiohydrolases is limited by slow dissociation and that cellobiohydrolases may stall in their processive movement due to obstacles on the substrate surface and the low dissociation rate (46, 47, 48, 49, 50). In such case, a reduction in the rate of dissociation, *k*_*off*_, or the processivity number, n, will lead to a decrease in V_max_/E_0_ as the maximal velocity of cellobiohydrolase may be approximated as V_max_/E_0_ ≈ n *k*_*off*_E_0_ (51). The decrease in V_max_ for *Tr*Cel7A on oxidized cellulose compared with nonoxidized cellulose may thus partly be due to a decrease in the processive movement of the cellobiohydrolase. However, our data showed similar processivity numbers of *Tt*Cel7A and *Mh*Cel7A on the two substrates, suggesting that other factors may also be contributing causes for the decrease in V_max_ on oxidized cellulose compared with nonoxidized cellulose.

Based on molecular dynamics simulation, LPMOs have been suggested to synergize with glycoside hydrolases by creating more solvent-accessible ends for glycoside hydrolases (19). The results in Figure 1 do not suggest a synergistic effect between reducing-end cellobiohydrolases and C1-oxidizing LPMOs, and the reduction in *k*_*on*_ on oxidized cellulose compared with nonoxidized cellulose indicates that the oxidized ends are poor attack sites for GH7 cellobiohydrolases. The oxidative activity of C1-oxidizing LPMOs thus effectivity reduces the amount of available good stack sites for reducing-end cellobiohydrolases.

Previous studies have shown that cellobiohydrolases may initiate hydrolysis internally in cellulose strands and hence use an endo-processive mode of action (49, 52, 53). As the extent of the impeding effect of C1-oxidations differed for the three cellobiohydrolases, one may speculate that the cellobiohydrolases differ in their propensity for endo-lytic attacks and hence may be more or less affected by changes to the chain ends. The results in Figure 6 showed that all three cellobiohydrolases had low activity against the endo-cellulase specific substrate, AZCL-HE-cellulose. The data showed only moderate differences in AZCL-HE-cellulose activity of the three cellobiohydrolases, which did not correlate with the degree of the impeding effect. This suggests that the extent to which the cellobiohydrolases were impeded by the oxidations could not be explained by differences in potential auxiliary endo-lytic activity.

The data presented here showed that oxidations led to reduced activity of all the cellobiohydrolases and that two out of three cellobiohydrolases were significantly impeded by the LPMO while one was unaffected or slightly boosted by the LPMO. This suggests that the well-known promoting effect of C1-oxidizing LPMOs on cellulolytic cocktails (2, 54) is not due to a direct promotion of the turnover of GH7 cellobiohydrolases. This is in line with the results of a recent study from our lab in which we suggested that the favorable effect of the LPMO predominantly reflected direct activation of endoglucanases and not the generation of new attack sites for cellobiohydrolases (23). We imagine that these results may be relevant for optimization of cellulolytic cocktails.

Detailed biochemical analyses of the cooperation between a C1-oxidizing LPMO and three GH7 cellobiohydrolases from different fungi revealed distinct differences in synergistic effects between the LPMO and the three cellobiohydrolases. One out of three cellobiohydrolases was severely impeded, one was moderately impeded, while the last was unaffected or slightly boosted by the LPMO.

Our results showed that the impeding effect is attributable to the introduction of oxidations on the cellulose surface, which reduced the maximal velocity and rate of substrate complexation of the cellobiohydrolases. The activity of C1-oxidizing LPMOs is thus effectively reducing the amount of available good attack sites for cellobiohydrolases. The severity of these effects varied considerably among the cellobiohydrolases, which may be relevant to consider when optimizing LPMO-enriched cellulolytic cocktails.

## Experimental procedures

### Enzymes

*T. terrestris* AA9E (*Tt*AA9, previously *Tt*GH61E) was provided as broth from Novozymes (Denmark) and purified and copper loaded as described elsewhere (55). *T. reesei* Cel7A (*Tr*Cel7A), *M. heterothallica* Cel7A (*Mh*Cel7A), and *T. terrestris* Cel7A (*Tt*Cel7A) were expressed in *Aspergillus oryzae* as described elsewhere (56). *Tr*Cel7A and *Mh*Cel7A were purified from the fermentation broths by hydrophobic interaction chromatography followed by ion-exchange chromatography on an ÄKTA system (GE Healthcare) as described elsewhere (57). *Tt*Cel7A was purified by hydrophobic interaction chromatography as for *Tr*Cel7A and *Mh*Cel7A, followed by size-exclusion chromatography on a HiLoad Superdex 75 pg column (GE Healthcare) equilibrated with 25 mM MES, pH 6.0. Next, the sample was purified by ion-exchange chromatography on a 26 ml SOURCE 15S column equilibrated with 50 mM acetate buffer pH 3. The sample was eluted with a linear gradient from 100 to 200 mM sodium chloride for 1.5 column volumes followed by 1.5 column volumes of 300 mM sodium chloride. The samples were purified to apparent homogeneity on a NuPAGE 4–12% Bis-Tris SDS-PAGE (Fig. S6). The enzyme concentrations were determined based on UV absorption and theoretical extinction coefficients (58) of 86,760 M^−1^ cm^−1^ (*Tr*Cel7A), 88,250 M^−1^ cm^−1^ (*Mh*Cel7A), 94,780 M^−1^ cm^−1^ (*Tt*Cel7A), *Tt*AA9E M^−1^ cm^−1^ (58,120). *Aspergillus niger* β-glucosidase was purchased from Megazyme.

### Substrates

Avicel PH101 (Sigma-Aldrich) was washed four times in MilliQ water and three times in 50 mM acetate buffer pH 5.0 (henceforth called standard buffer). Phosphoric acid swollen cellulose (PASC) was produced from Avicel PH101 (Sigma-Aldrich) as described previously (59). The PASC suspension was divided into two stocks; one was washed three times in standard buffer and resuspended in standard buffer, this stock is referred to as PASC, the other stock was treated with CuSO_4_ as described below and referred to as oxidized PASC.

### Oxidation of insoluble reducing ends in PASC by Cu(II)

The reducing ends in PASC were oxidized with CuSO_4_ as described elsewhere (41). In short, 17 g/L PASC was incubated with 2.8 mM CuSO_4_, 46 mM bicinchoninic acid (BCA), and 170 mM Na_2_CO_3_ in 100 ml suspension. The mixture was incubated at room temperature in an orbital shaker at 5 rpm for 30 days. Hereafter a sample was kept for quantification of the oxidation level, and the PASC was washed ten times in 2 L water and three times in standard buffer using a vacuum pump and resuspended in standard buffer. To verify that the oxidation method solely oxidized the C1-position of the glucose moieties and that it did not introduce chain breakages, samples of 500 μM cellotetraose were incubated 30 days at room temperature with 2.8 mM CuSO_4_, 46 mM BCA, and 170 mM Na_2_CO_3_. The products were analyzed as described below for the PASC preparation and by high-performance anion exchange chromatography with a pulsed amperometric detection (HPEAC-PAD) as described elsewhere (60).

### Quantification of the level of oxidation

When PASC was mixed with CuSO4, BCA, and Na_2_CO_3_, a violet color of the Cu(I)BCA_2_ complex developed, which could be detected at 560 nm. This indicated that Cu(II) was reduced to Cu(I) while the reducing ends in the cellulose were oxidized (61). The level of oxidation was determined by colorimetric quantification of Cu(I)BCA_2_ in the presence of L-serine by two approaches: (1) by quantifying Cu(I)BCA_2_ in the supernatant of the sample, and (2) by quantifying the remaining reducing ends in the PASC and comparing with a nontreated sample. For both approaches, samples of 500 μl oxidized PASC and 500 μl nonoxidized PASC were used. The samples were centrifuged, and 200 μl supernatant was used to quantify Cu(I)BCA_2_ as described elsewhere (61), and the pellet was kept for quantification of remaining reducing ends. (1) The concentration of glucose-equivalent reducing ends was quantified based on a linear calibration curve with six concentrations of glucose in the range of 0–0.125 mM. The quantification of the level of oxidation was based on the theoretical amount of reducing ends in a nontreated PASC sample. This was estimated using an average degree of polymerization (DP) of Avicel of 218 (62), as 1/218 of the anhydroglucosyl units. (2) The pellets were washed 20 times with 1 ml MilliQ water by centrifugation and resuspension. The concentration of insoluble reducing ends was quantified as described elsewhere (61). A calibration curve with varying loads of PASC was linear in the range 0–5 g/L. Using this data, we plotted the measured concentration of reducing ends as a function of the theoretical concentration of reducing ends as described above and found a slope of 0.92, indicating that the method could be used to estimate the number of insoluble reducing ends. The samples were diluted ten times in MilliQ water to reach 1.7 g/L. The level of oxidation was determined from the difference in reducing ends in the nontreated sample and the reducing ends in the Cu(II) treated sample. All experiments were performed in triplicates.

### Synergy experiments

The activity of each cellobiohydrolase (*Tr*Cel7A, *Tt*Cel7A, or *Mh*Cel7A) and *Tt*AA9 was assessed alone and in combinations (Cel7A + *Tt*AA9). In experiments with mixtures of Cel7A and AA9, the total enzyme concentration was 1 μM, while the ratio of the two components was varied systematically. In experiments with only Cel7A or *Tt*AA9, the enzyme concentration varied from 0 to 1 μM. All samples were incubated with 10 g/L Avicel, 1 mM ascorbic acid in 50 mM acetate buffer at pH 5 in 96-well plates in a Thermomixer C (Eppendorf) at 50 °C, 1100 rpm, for 3 h. Hereafter, the plates were centrifuged at 3000*g* for 3 min, and 50 μl supernatant was mixed with 1.7 U/ml β-glucosidase from *A. niger* (Megazyme) in a total volume of 150 μl. The samples were incubated at 50 °C, 300 rpm, for 20h. Hereafter, 65 μl sample was transferred to a PCR plate, and the concentration of reducing sugars was determined using the p-hydroxybenzoic acid hydrazide (PAHBAH) assay as described earlier (63). The concentration of reducing sugars was determined based on linear calibration curves with eight different concentrations of glucose in the range 0–1 mM in standard buffer, which was included in each plate.

### Michaelis–Menten assay

Samples of varying loads of PASC or oxidized PASC in the range 0–8 g/L and 100 mM cellobiohydrolase (*Tr*Cel7a, *Tt*Cel7A, or *Mh*Cel7A) were incubated in 96-well plates in a Thermomixer C (Eppendorf) at 50 °C, 1100 rpm, for 1h. Hereafter, the plates were centrifuged at 2000*g* for 3 min, and 65 μl supernatant was transferred to a PCR plate. The concentration of reducing sugars was determined using the PAHBAH assay as described earlier (63). The concentration of reducing sugars was determined based on linear calibration curves with eight different concentrations of cellobiose in the range 0–1 mM in standard buffer, which were included in each plate.

### Product profile

Samples of 4 g/L PASC or oxidized PASC and 100 nM cellobiohydrolase (*Tr*Cel7A, *Tt*Cel7A, or *Mh*Cel7A) in a final volume of 250 μl were incubated in 96-well plates in a Thermomixer C (Eppendorf) at 50 °C, 1100 rpm, for 1h. Hereafter, the plates were centrifuged at 2000*g* for 3 min, and 100 μl supernatant was mixed with 200 μl 0.1 M NaOH. The samples were analyzed by HPAEC on an ICS5000 system equipped with a pulsed amperometric detector (PAD) (Dionex), a CarboPac PA1 guard column (4 × 50 mm), and an analytical CarboPac PA1 column (4 × 250 mm). The analytes were eluted at 1.3 ml min^−1^ at 30 °C, and the initial condition was 100% 0.1 M NaOH (eluent A). A linear gradient was applied, increasing the proportion of eluent B (0.5 M NaOAc in 0.1 M NaOH) to 15% B after 7.5 min. A steep linear gradient was applied to reach 85% B after 7.75 min. Hereafter, a linear gradient was applied to reach initial conditions after 8 min. These conditions were kept for 4.5 min. The products were assigned based on standard solutions containing glucose, cellobiose, and cello-oligomers with a degree of polymerization (DP) up to four (Megazyme) and oxidized standards generated by base-catalyzed oxidation of the native cello-oligomers with iodine as described elsewhere (60).

### Biosensor measurements

Real-time measurements were performed with a cellobiose dehydrogenase (CDH) biosensor. The biosensors were prepared as described previously (31). The measurements were performed with 1 g/L PASC or oxidized PASC and addition of 100 nM *Tr*Cel7A, *Tt*Cel7A, or *Mh*Cel7A in a final volume of 4 ml.

### Real-time measurement of complexation

Measurements of complexation were conducted in a Jasco FP-8200 spectrofluorometer equipped with a custom-made injection inlet to the quartz cuvette and a thermostat set to 25 °C and magnetic stirrer (Jasco STR-811) as described earlier (35).

The method relies on changes in the fluorescence emission at 328 nm when cellobiohydrolases bind the substrate. The fluorescence signal was monitored (excitation 295 nm, emission 328 nm) from 2 ml of 250 nM cellobiohydrolase with vigorous stirring. After 5 s, the signal was stable, and 100 μl PASC or oxidized PASC was added with a syringe, and the changes in fluorescence were recorded over 200 s. Hereafter a full spectrum (excitation 295 nm, emission 310–500 nm) was recorded. These two experiments were repeated with six different loads of PASC and oxidized PASC (in the range 7.5 mg/L-200 mg/L) for each of the three cellobiohydrolases. Full spectra (excitation 295 nm, emission 310–500 nm) were also recorded with bovine serum albumin (BSA) with all the tested substrate loads (both PASC and oxidized PASC) as controls to assess the loss in emission associated with light scattering from the substrates as described earlier (35). The loss of emission was identical for the two substrates (Fig. S7).

### Activity against AZCL-HE-cellulose

Endolytic activity was determined using the insoluble substrate azurine-cross-linked cellulose (AZCL-HE-Cellulose) (Megazyme). The substrate load was 3 g/L, and the enzyme concentrations were 5 μM for the cellobiohydrolases and 0.1 μM for the endoglucanase. The samples were incubated in a 96-well plate in a Thermomixer C (Eppendorf) at 50 °C, 1100 rpm, for 2 h. Hereafter the plate was centrifuged, and 100 μl supernatant was transferred to a 96-well plate, and the absorbance was measured at 595 nm.

## Data availability

The authors confirm that the data supporting the findings of this study are available within the article and its supporting information.

## Supporting information

This article contains supporting information.

## Conflicts of interest

The authors declare the following conflicts of interest: Nanna Røjel, Trine H. Sørensen, Kim Borch, and Brett McBrayer work for Novozymes A/S, a major manufacturer of industrial enzymes.
