# Supplementary material for: A comparative biochemical investigation of the impeding effect of C1-oxidizing LPMOs on cellobiohydrolases
Source: J Biol Chem. 2021 Mar 3;296:100504. doi: 10.1016/j.jbc.2021.100504 (PMC8047454; doi:10.1016/j.jbc.2021.100504)
Supplement: Figures S1 to S7 [file mmc1.pdf]

Supporting information

A comparative biochemical investigation of the impeding effect of C1-oxidizing LPMOs on cellobiohydrolases

Malene Billeskov Keller[1], Silke Flindt Badino[2], Nanna Røjel[2,†], Trine Holst Sørensen[3], Jeppe Kari[2], Brett McBrayer[4], Kim Borch[3], Benedikt M. Blossom[1,*], and Peter Westh[2,*]

[1]Department of Geosciences and Natural Resource Management, University of Copenhagen, 23 Rolighedsvej, Frederiksberg, DK-1958, Denmark

[2]Department of Biotechnology and Biomedicine, Technical University of Denmark, 221 Søltofts Plads, Kgs. Lyngby, DK-2800, Denmark

[3]Novozymes A/S, 2 Biologiens Vej, Kgs. Lyngby, DK-2800, Denmark

[4]Novozymes, Inc., 1445 Drew Ave, Davis, CA 95618, USA.

[*]Corresponding authors

Peter Westh

Phone: (+45) 3091 5488

Email: petwe@dtu.dk

Fax: (+45) 4588 4922

Benedikt M. Blossom

Phone: (+45) 3533 0327

Email: kbm@ign.ku.dk

[†]Current address: Novozymes A/S, 36 Krogshøjvej, Bagsværd, DK-2880, Denmark

Running title: Impeding effect of LPMOs on cellobiohydrolases

List of materials included:

Figure S1: High-performance anion-exchange chromatogram of oxidation product of celloteraose after oxidation with $CuSO_4$
Figure S2: Inverse Michaelis–Menten experiments on PASC and oxidized PASC
Figure S3: Ratio of cellobiose/(glucose + cellotriose) produced on PASC and oxidized PASC
Figure S4: Real-time fluorescence data for complexation of PASC and oxidized PASC
Figure S5: Binding curves from real-time fluorescence experiments
Figure S6: SDS page of cellobiohydrolases and LPMO
Figure S7: Emission spectra for BSA control experiments

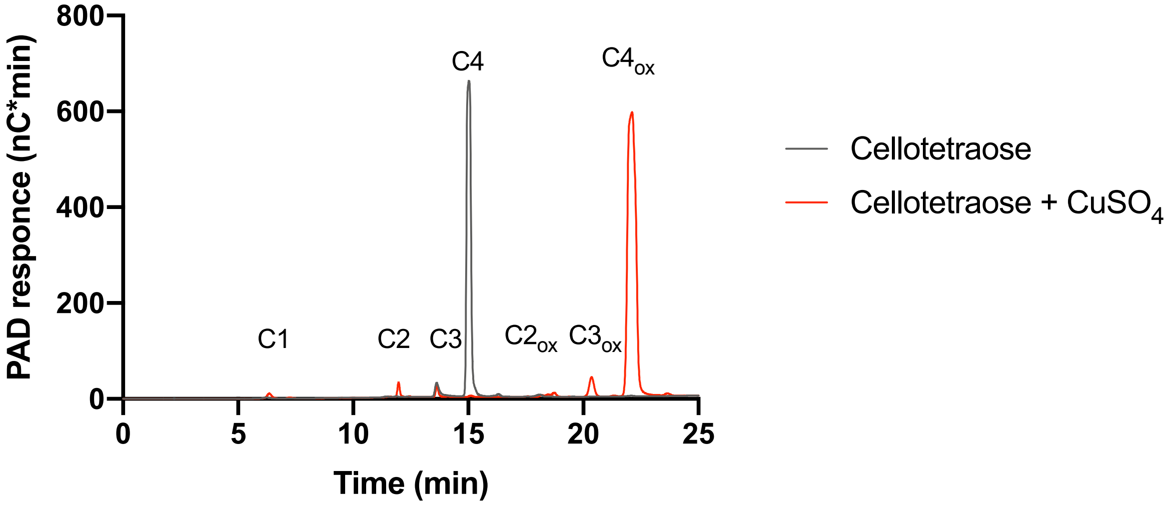

Figure S1. High-performance anion-exchange chromatography (HPAEC) elution pattern of cellotetraose (grey) and a sample of cellotetraose incubated with 2.8 mM $CuSO_4$, 170 mM Na_2CO_3, and 46 mM bicinchoninic acid (BCA) for 30 days at room temperature (red). The peaks are assigned based on standards of glucose (C1), cellobiose (C2), cellotriose (C3), cellotetraose (C4) and oxidized standards of cellobionic acid ($C2_{ox}$), cellotrionic acid ($C3_{ox}$), and cellopentaonic acid ($C4_{ox}$). The experiment verified that the treatment with $CuSO_4$ oxidized the sample and that the treatment did not introduce significant chain breakage.

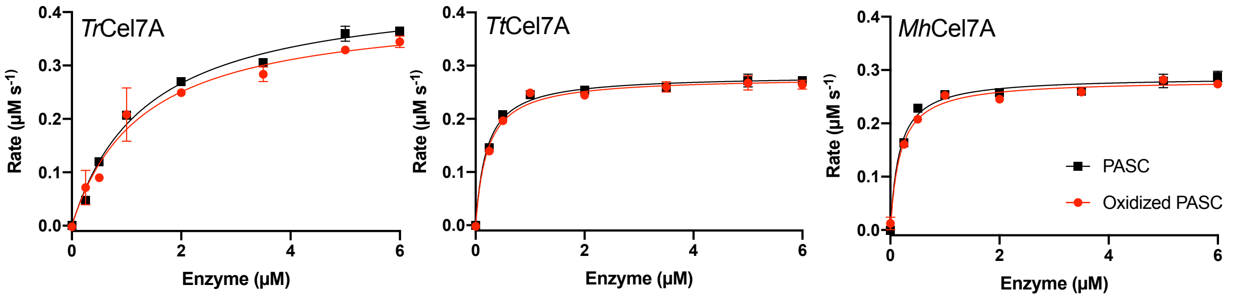

Figure S2. Rate of *Tr*Cel7A, *Tt*Cel7A, and *Mh*Cel7A against 0.5 g/L PASC (black squares) or oxidized PASC (red circles) at varying concentrations of enzyme for 1h experiments. Symbols are averages of triplicate measurements and error bars represent standard deviations (s.d.). Lines are best fit of a Michaelis-Menten type equation.

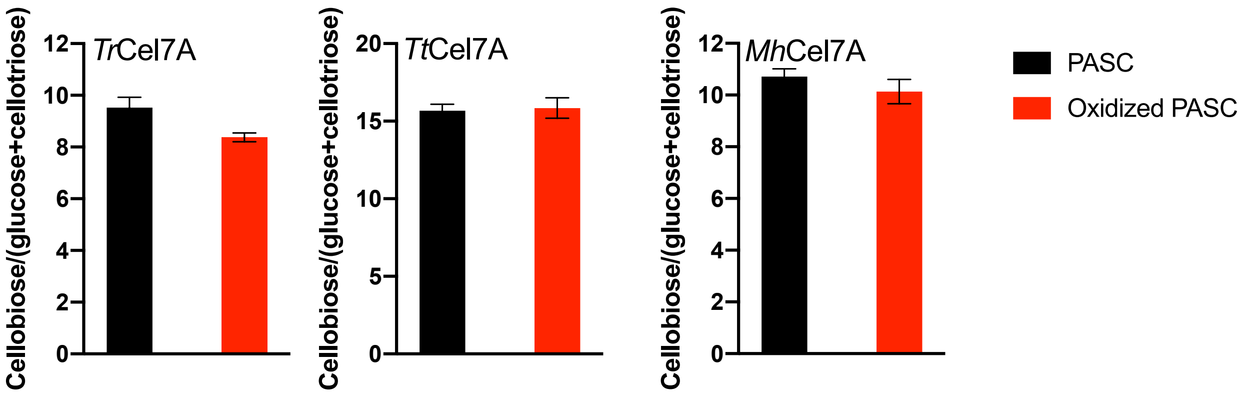

Figure S3. Ratio of production of cellobiose/(glucose + cellotriose) by 100 nM *Tr*Cel7A, *Tt*Cel7A, and *Mh*Cel7A against 2 g/L PASC (black bars) or oxidized PASC (red bars). Error bars represent propagated standard deviations (SD) of triplicate measurements.

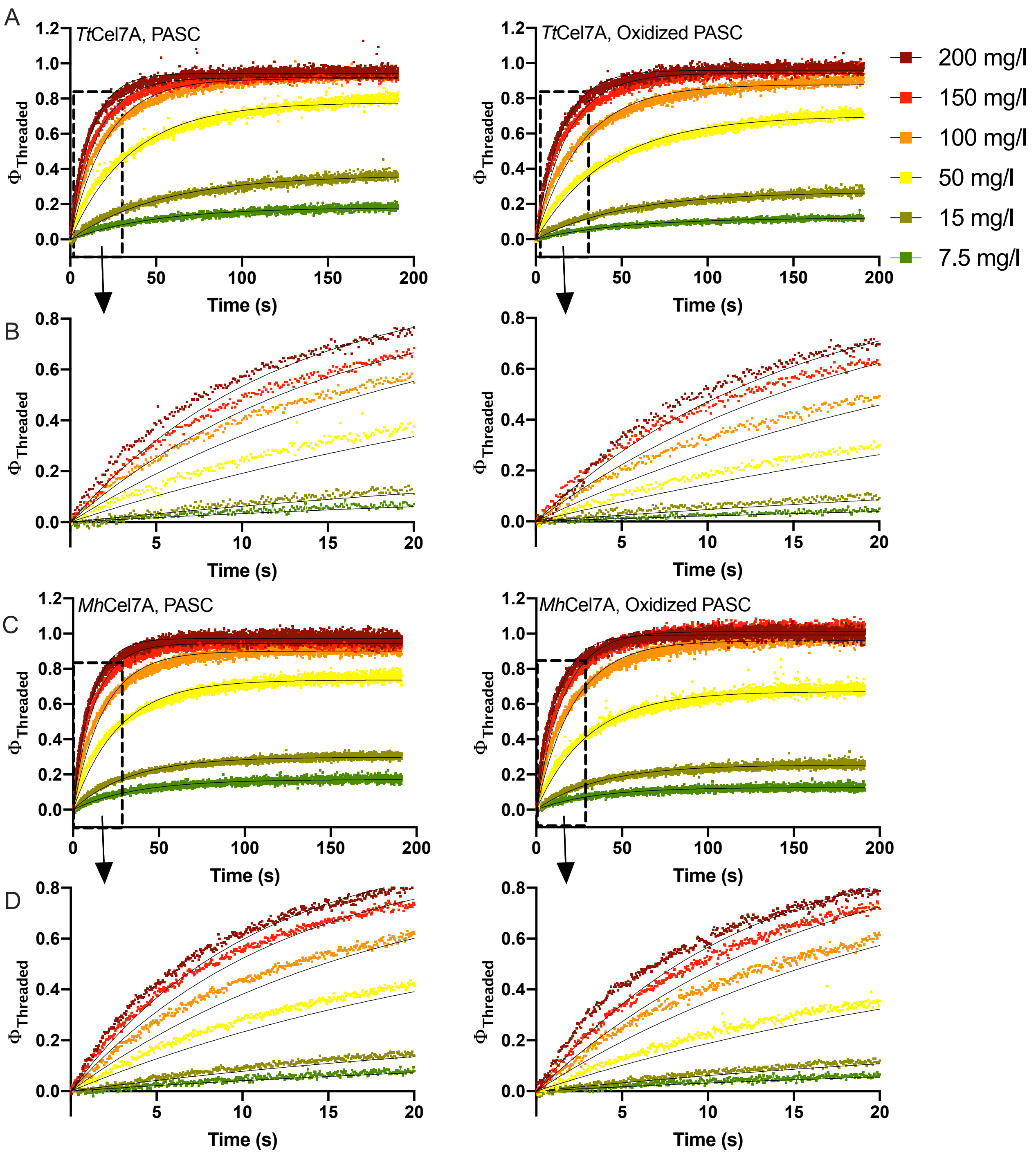

Figure S4. Real-time fluorescence data for the complexation of PASC (left) and oxidized PASC (right) of *Tt*Cel7A (A,B) and MhCel7A (C,D) represented as the fraction of threaded enzymes, $\Phi_{threaded}$ against time. The lines represent the best fit to an exponential function of the type,

$F_x(t)=F_{eq}(1-e^{-kt})$. B. Enlargements (B,D) of data from 0-20s, as indicated by the dotted boxes in A and C.

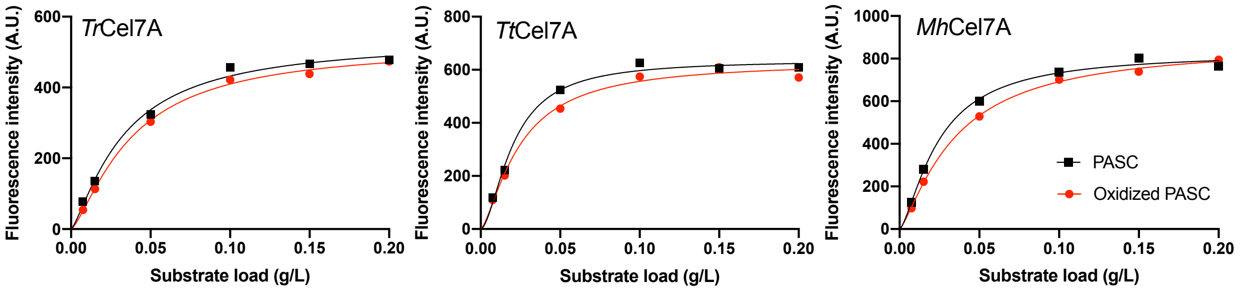

Figure S5. Binding curves from real-time fluorescence experiments. The equilibrium fluorescent signal, F_{eq} is plotted against the substrate load PASC (black squares) or oxidized PASC (red circles). Lines are best fit of the Hill equation, $f(x)=(y_{max}x^h)/(k_d{}^h+x^h)$.

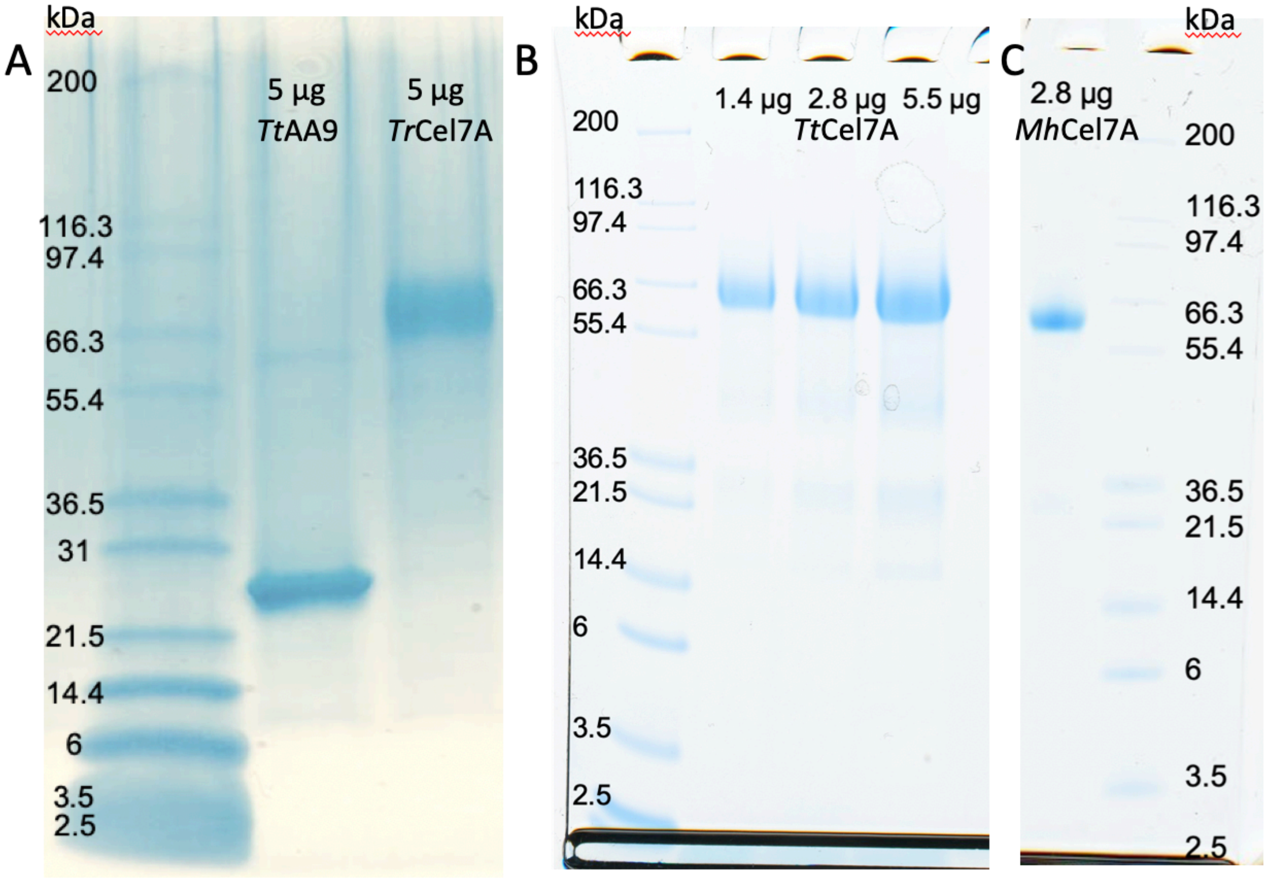

Figure S6. SDS NuPAGE 4-12% BisTris gel. The proteins were stained with InstantBlue protein stain, Cromassie Brilliant Blue (Expedeon). The amount of protein on the gel in noted above each band. A. *Tr*Cel7A and *Tt*AA9. B. *Tt*Cel7A, C. *Mh*Cel7A

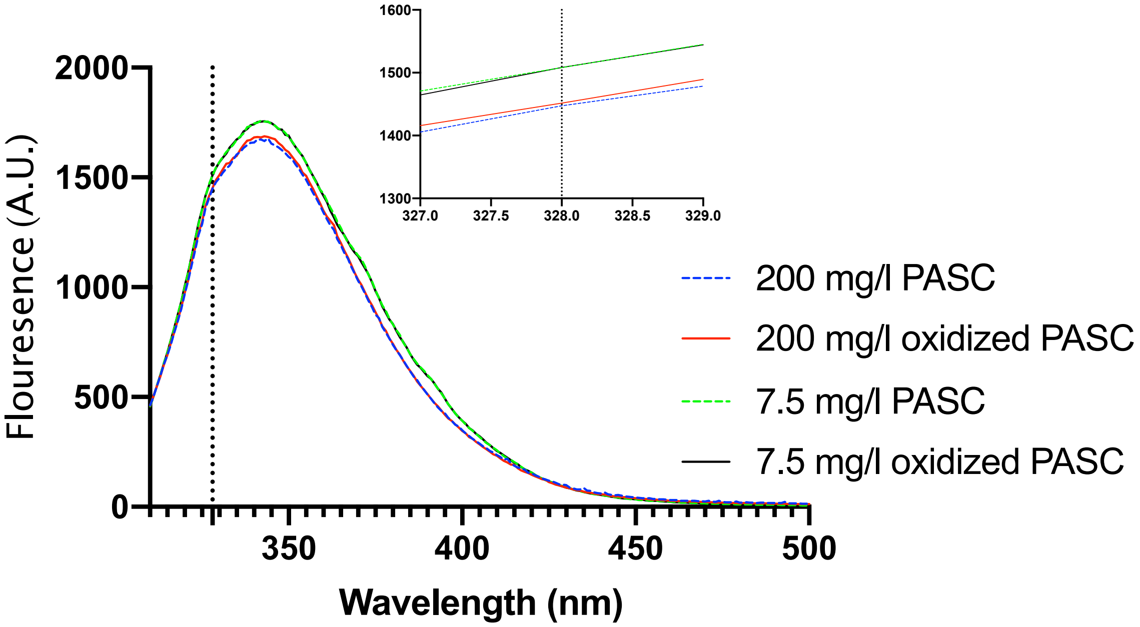

Figure S7. Emission spectra for BSA control experiments. If we assume that the fluorescence emission from a non-binding protein like BSA is independent of the presence of PASC particles, possible changes may be ascribed to scattering light scattering (of either excitation or emission). We indeed found small changes that scaled with the substrate load. For clarity, the figure only shows two substrate loads, 7.5 mg/l PASC (green dotted lines), 7.5 mg/l oxidized PASC (black lines), 200 mg/l PASC (blue dotted lines), and 200 mg/l oxidized PASC (red lines). The remaining spectra with intermediate substrate loads fell between these two extremes. The ordinate shows fluorescence emission in arbitrary units. It appears from the enlargement in the inset that fluorescence emission at 328 nm (which was used in the binding analysis) fell by some 50 units from the lowest to the highest substrate load. This substrate-load dependent change was assumed to be transferable to the cellulase measurements and incorporated into the data analysis.
